# Understanding the impact of different bioprocess conditions on monoclonal antibody glycosylation in CHO cell cultures through experimental and computational analyses

**DOI:** 10.1186/1753-6561-9-S9-O6

**Published:** 2015-12-14

**Authors:** Si N Sou, Christopher Sellick, Ken Lee, Alison Mason, Sarantos Kyriakopoulos, Karen M Polizzi, Cleo Kontoravdi

**Affiliations:** 1 Department of Chemical Engineering; 2 Department of Life Sciences; 3 Centre for Synthetic Biology and Innovation, Imperial College London, London SW7 2AZ, U.K; 4 Cell Culture and Fermentation Sciences, MedImmune, Granta Park, Cambridge, CB21 6GH, U.K

## Background and Novelty

Key Words: Monoclonal antibody glycosylation, stable & transient gene expression, mild hypothermia, mathematical modelling, flux balance analysis

With positive outcomes from medical treatments, monoclonal antibodies (mAbs) are the best-selling biologics in the pharmaceutical market to date. The medical value and safety of these molecules have been sometimes reported to be impacted by the carbohydrate structures that are attached to the N-linked glycosylation site on the constant region of the mAb, such as rituximab in which the glycan pattern on its Fc-region determines the function of rituximab in eliciting immune response against diseases such as leukemias and autoimmune disorders that are characterised by abnormal production of dysfunctional B cells [1]. It has also been reported that different bioprocess conditions during recombinant mAb production directly impact glycan compositions [2,3], although the mechanism behind this change is not fully understood. This lack of understanding limits process design and optimisation efforts. Herein, we examine the effect of mild hypothermic conditions. Mild hypothermia during cell culture (e.g. temperature shift from 36.5°C to 32°C) is commonly employed to increase the specific recombinant product productivity (qP) [4,5]. Cells are commonly cultured at physiological temperature during the start of the production stage bioreactor step until desired viable cell density is obtained; the temperature is then lowered to mild hypothermic levels (ranges from 28°C to 34°C), where the lower rates of cell growth are typically compensated for by an the increase in qP. In this study, we investigate experimentally the effect of mild hypothermia (32°C) on mAb N-linked glycosylation, using flux balance analysis and mathematical modelling to identify resulting differences in cell metabolism. A mathematical model that mechanistically and quantitatively has been constructed to describe four different elements: 1. CHO cell behaviour and metabolism; 2. mAb synthesis; 3. Nucleotide sugar donor (NSD) metabolism; and 4. mAb Fc N-linked glycosylation profiles, before and after the induction of mild hypothermia. We believe that this is the first quantitative model that relates mild hypothermia to the four elements mentioned above. As the model aids understanding of the way bioprocess conditions affect product quality, it also provides a platform for bioprocess design, control and optimisation in industry and helps the implementation of the Quality by Design principles.

## Experimental Approach

Firstly CHO culture performance was measured with respect to the following: 1. cell growth, nutrient and metabolite concentrations; 2. mAb production at transcriptional, translational and secretion levels through quantitative real-time PCR, western blotting and affinity chromatography; 3. N-linked glycan profiles of the product via a LabChip method, as well as 4. mRNA and protein expressions of N-linked glycan related enzymes, in all bioprocess conditions investigated - physiological and mild hypothermic conditions. To better understand CHO cell metabolism in all conditions, intracellular carbon fluxes were estimated using flux balance analysis [6] that was constrained with our experimental exometabolite data. Next we related the effect of each process condition with mAb glycosylation with the help of a modular mathematical model that was adapted from previous models [7-9]. This describes CHO cell growth, nutrient metabolism, NSD metabolism, mAb synthesis and Golgi N-linked glycosylation.

## Results and Discussion

**Figure 1:**
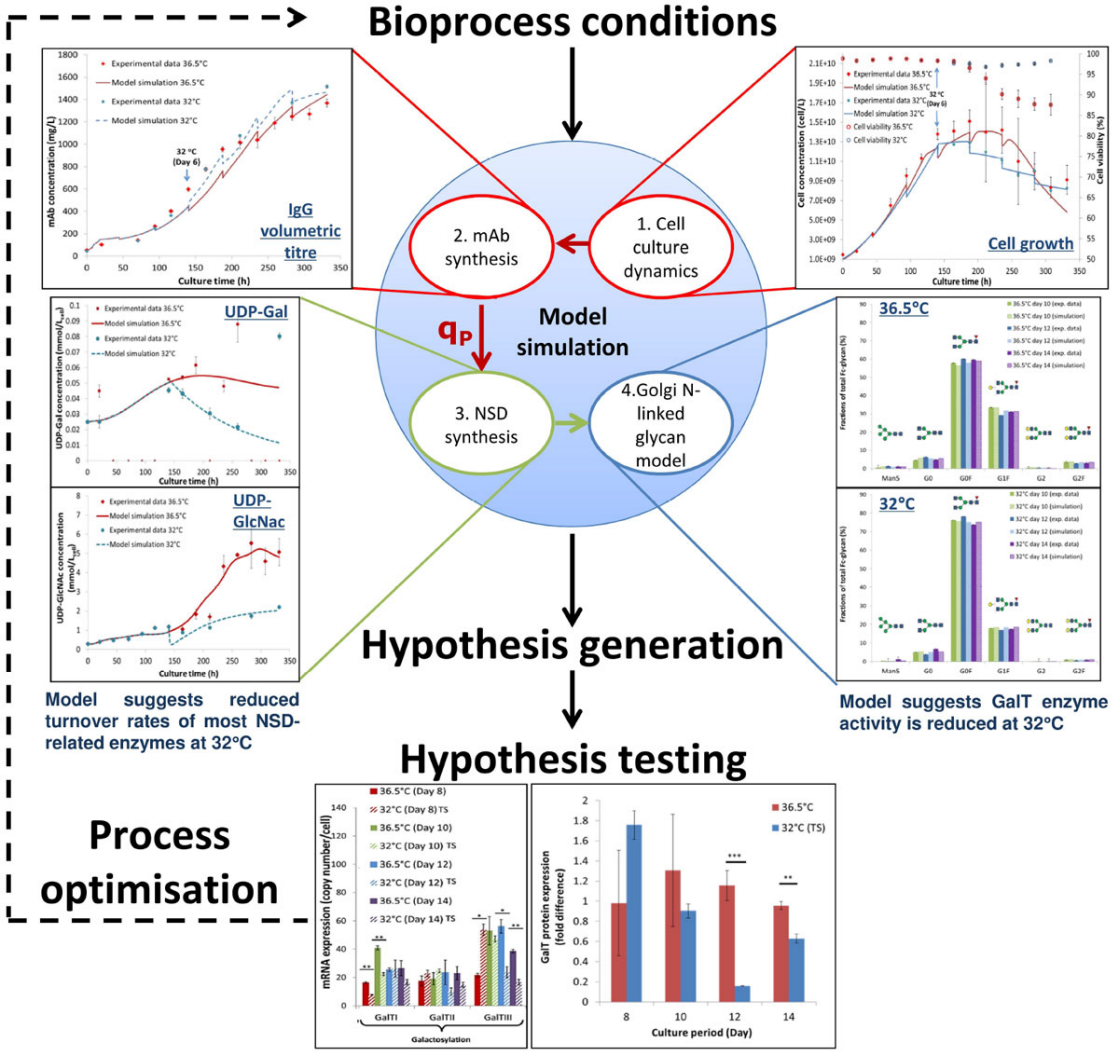
**Model framework and model simulations of CHO cells cultured in mild hypothermic conditions**.

CHO cells cultured in mild hypothermic conditions exhibited prolonged cell viability, lower rates of nutrient and NSD metabolism, as well as a 20% increase in the specific mAb productivity (qmab). In addition, glycan profiling showed limitation in terminal galactosylation on the mAb Fc region at 32°C. Flux balance analysis was used to identify factors that contributed to changes observed in mAb Fc-glycosylation under mild hypothermia. The results demonstrated reduced carbon fluxes towards nucleotide, NSD and lipid synthesis at 32°C [10]. The modular mathematical model represented the time-course data well, as well as reproducing the N-linked glycosylation profile of mAb produced under mild hypothermia. Through parameter estimation, the model suggested that higher qmAb observed is accompanied by increased transcription, translation and transport rates of mAb molecules. Importantly, estimation results suggested that the rates of NSD production and galactosyltransferase (GalT) expression were reduced at 32°C. This was then confirmed by the experimental measurements of UDP-Gal concentration and GalT expression levels (Figure 1), thus closing the loop between experimental and computational systems.

## Conclusions

Results from both experimental and computational studies show that reduced NSD production and lower expression of N-linked glycosyltransferases are the main causes of glycan differences in cultures grown under mild hypothermia. As a result, feeding of nucleosides and other carbon sources, together with manipulation of the expression of glycosyltransferase can be employed to improve mAb glycosylation. In this way, quality can be built into products and processes to satisfy following the Quality by Design paradigm.

## Acknowledgements

SNS thankfully acknowledges the Biotechnology and Biological Sciences Research Council and Bioprocessing Research Industry Club for her studentship. The financial contribution of MedImmune plc is gratefully acknowledged. The authors thank Ioscani Jimenez del Val and Philip M Jedrzejewski for their help in the model development, as well as Kalpana Nayyar, Andrew Smith and Neil Birkett for their assistance in glycan, mAb titre analyses and mAb purification, respectively. KMP and CK thank Research Councils U.K. for their fellowships. CK thanks Lonza Biologics for their financial support.
